# Chemotherapy induced cardiomyopathy: an overview, imaging features, and future prospective

**DOI:** 10.1186/1470-7330-15-S1-P21

**Published:** 2015-10-02

**Authors:** D Divito, M Bondin, SK Kirschner, J Stojanovska, E Ibrahim, L Frank

**Affiliations:** 1 University of Texas Medical Branch, 301 University Boulevard, Galveston, TX, USA

## Learning objectives

To review the spectrum of imaging findings of chemotherapy- induced cardiomyopathy in correlation with most common cytotoxic drugs and regimens.

## Content organisation

Cardio toxic effect of chemotherapy is a well-recognised problem in cancer patients. Cardio toxicity depends on multiple predisposing factors, specific components of the chemotherapy regimen, length of treatment, and dosage.

We will present the spectrum of most common cardiotoxic chemotherapy agents and their combinations, specific effects on the myocardium, and imaging features of cardiomyopathies induced by chemotherapy.

We will review pathophysiology of chemotherapy induced cardiomyopathy including:

• Dose dependent cardiomyopathy

• Predisposing conditions –diabetes, presence of coronary artery disease, age.

• Potential reversibility

We will discuss imaging characteristics of chemotherapy induced cardiomyopathy

• Imaging modalities (Echocardiography, Cardiac MR, and MUGA)

• Importance of monitoring cardiac function during and after treatment

• Distribution of late Gadolinium enhancement (LGE)

• Emerging technologies for early diagnosis of cardiomyopathy in cancer patients

## Conclusions

Chemotherapy induced cardiomyopathy is a common problem among cancer patients, increasing long term morbidity and mortality and often leading to disability. Patients receiving chemotherapy treatment, particularly cardio toxic agents, should be routinely assessed for cardiac function to diagnose cardiomyopathy during the early phase of treatment and to prevent development of irreversible heart failure.

